# Investigating Molecular Signatures Underlying Trapeziometacarpal Osteoarthritis Through the Evaluation of Systemic Cytokine Expression

**DOI:** 10.3389/fimmu.2021.794792

**Published:** 2022-01-20

**Authors:** Anusha Ratneswaran, Jason S. Rockel, Daniel Antflek, John J. Matelski, Konstantin Shestopaloff, Mohit Kapoor, Heather Baltzer

**Affiliations:** ^1^ Hand Program, Schroeder Arthritis Institute, University Health Network, Toronto, ON, Canada; ^2^ Division of Orthopedics, Osteoarthritis Research Program, Schroeder Arthritis Institute, University Health Network, Toronto, ON, Canada; ^3^ Krembil Research Institute, University Health Network, Toronto, ON, Canada; ^4^ Biostatistics Research Unit, University Health Network, Toronto, ON, Canada; ^5^ Department of Surgery and Laboratory Medicine and Pathobiology, University of Toronto, Toronto, ON, Canada; ^6^ Division of Plastic and Reconstructive Surgery, University of Toronto, Toronto, ON, Canada

**Keywords:** inflammation, osteoarthritis, trapeziometacarpal osteoarthritis, cytokine, molecular factors

## Abstract

**Purpose:**

Non-operative management of trapeziometacarpal osteoarthritis (TMOA) demonstrates only short-term symptomatic alleviation, and no approved disease modifying drugs exist to treat this condition. A key issue in these patients is that radiographic disease severity can be discordant with patient reported pain, illustrating the need to identify molecular mediators of disease. This study characterizes the biochemical profile of TMOA patients to elucidate molecular mechanisms driving TMOA progression.

**Methods:**

Plasma from patients with symptomatic TMOA undergoing surgical (n=39) or non-surgical management (n=44) with 1-year post-surgical follow-up were compared using a targeted panel of 27 cytokines. Radiographic (Eaton-Littler), anthropometric, longitudinal pain (VAS, TASD, quick DASH) and functional (key pinch, grip strength) data were used to evaluate relationships between structure, pain, and systemic cytokine expression. Principal Component Analysis was used to identify clusters of patients.

**Results:**

Patients undergoing surgery had greater BMI as well as higher baseline quick DASH, TASD scores. Systemically, these patients could only be distinguished by differing levels of Interleukin-7 (IL-7), with an adjusted odds ratio of 0.22 for surgery for those with increased levels of this cytokine. Interestingly, PCA analysis of all patients (regardless of surgical status) identified a subset of patients with an “inflammatory” phenotype, as defined by a unique molecular signature consisting of thirteen cytokines.

**Conclusion:**

Overall, this study demonstrated that circulating cytokines are capable of distinguishing TMOA disease severity, and identified IL-7 as a target capable of differentiating disease severity with higher levels associated with a decreased likelihood of TMOA needing surgical intervention. It also identified a cluster of patients who segregate based on a molecular signature of select cytokines.

## Introduction

Osteoarthritis at the base of the thumb (Trapeziometacarpal Osteoarthritis [TMOA]), is a prevalent and painful condition (1). The etiology of TMOA is unknown, and pain is the main reason individuals seek medical attention (2). Though studies examining the occurrence of this specific condition are lacking, it is estimated that TMOA has a lifetime prevalence of approximately 10% (3, 4). This is highly variable between radiographic TMOA which has an estimated prevalence of 12-50%, and symptomatic TMOA which affects 1-16% of individuals (5). This joint site, in particular, is understudied compared to other osteoarthritic locations such as the knee. Historically, the TM joint has been grouped together with other hand joints in OA studies despite evidence supporting it as distinctly affected (6).

Risk factors for TMOA include age, obesity, heritability, repetitive occupational thumb use, ethnicity, and radial subluxation at the base of the thumb (in males) (1, 7–9). Preventative measures need further investigation, and subsequent meta-analyses. Currently, there are no pharmacological treatments which reduce the progression of TMOA, and early-stage non-operative management has typically shown only short-term symptomatic benefits (10–13). Patients who do not respond to non-operative therapy may opt to receive surgical intervention, often with lengthy recovery periods.

Radiographic TMOA severity is often discordant with patient reported pain and functional assessments (14–16). These inconsistencies illustrate the need for objective markers linking disease severity to clinical measures in order to detect the disease early, predict which patients are more likely to develop severe or rapidly progressing disease, evaluate treatment response, and develop probable treatment targets (17, 18). Yet, there are also no validated prognostic or diagnostic biomarkers of TMOA (19). While their current utility may be limited, a sensitive diagnostic tool which is able to identify TMOA early, and predict prognosis could benefit patients by reducing exposure to risk factors and helping start preventative strategies and therapies early as more efficacious regimes are identified. Identification of an objective marker or effective therapeutic target is contingent on a strong understanding of molecular mechanisms underlying disease progression. Profiling potential molecular or secretory signatures in a TMOA patient population can contribute to understanding their role within a disease-specific context and build a foundation for identifying these markers.

Cytokines are secreted signalling molecules that reflect active processes within the joint such as inflammation, cartilage synthesis and destruction, and bone remodeling, thereby having the ability to translate the intrinsic state of the joint proximally, or even systemically. Tissue damage and low level chronic inflammation in OA generates cytokines, which can alter joint tissue homeostasis directly [through the involvement in OA pathophysiology such as Interleukin(IL)-1B, IL-4, IL-6, IL-10, IL-13, TNFa] or indirectly through processes such as angiogenesis, chemotaxis, and inflammation (20, 21). A limitation which must be acknowledged is that it is largely unknown whether systemic levels of cytokines could reflect pathological processes in such a small joint. However, it has been reported that systemic cytokines are correlated to bone resorption in temporomandibular joint OA (22). Furthermore, CMC (TM) joints disproportionately impact the concentration of systemic OA biomarkers while joint size does not determine the contribution to systemic biomarker concentration (23). This suggests that these OA in these small joints can impact systemic molecular profiles.

In this study, we sought to determine molecular indicators which could classify TMOA patients based on clinical, biological and anthropometric factors. We evaluated whether circulating cytokines can distinguish symptomatic disease severity by comparing patients undergoing non-surgical management to those undergoing surgery (trapeziectomy), and provide a basis for communicating active processes occurring within the joint. We show that the cytokine IL-7 is capable of discriminating disease severity between these two groups. We also discovered that regardless of surgical status patients can be sub-classified into separate groups based on a distinct molecular signature of thirteen inflammatory cytokines indicating there may be different molecular phenotypes within this population.

## Materials and Methods

### Study Population

Symptomatic TMOA patients receiving non-surgical management (splinting, education, physiotherapy or standard operative intervention (trapeziectomy with/without ligament reconstruction and tendon interposition), followed the pipeline in **Supplementary Figure 1** (approved study #16-5759). Indications for surgery included: persistent pain that limits normal hand function as assessed by patient report and clinical parameters including limited range of motion, deformity, grip and pinch strengths; failure of non-surgical measures; and capacity to give informed consent. Treatment program was based on surgeon recommendation with patient involvement in decision making. Patients were excluded from the study if they had post-traumatic, crystalline arthritis or corticosteroid injections within the past three months. Patients with steroid injections were excluded due to the possible effects on systemic cytokines. They were excluded in both the surgical group and non-surgical group.

Plasma was collected at baseline (pre-treatment) for surgical and non-surgical patients, and post-surgical time points of 6 weeks, 3 months, 6 months and 1 year. Clinical characteristics (age, sex, BMI, and painful joint count were self-reported by the participant. Functional measures (key pinch, grip strength), were conducted in triplicate using a dedicated Jamar pinch-gauge and dynamometer (Sammons Preston) using the average to produce a final value. Participants were asked to indicate the overall intensity of their thumb pain from 0-100 using an electronic Visual Analogue Scale(VAS) as well as symptoms and function using the shortened Disability of the Arm, Shoulder and Hand- quick DASH, and Trapeziometacarpal Arthrosis Symptom and Disability Questionnaire- TASD) (24–27). The quick DASH is an abbreviated version of the DASH questionnaire that includes 11 of the original 30 items and is used to assess symptoms and function in the upper extremities. Responses for each item are indicated on a 5-point Likert scale ranging from no disability (1 point) to extreme disability (5 points). A summative score out of 100 (where 100 indicates greatest disability) was obtained by summing the value of responses, dividing by the number of completed items, subtracting 1, and then multiplying that value by 25 (28). The TASD also uses a 5-point Likert scale and has 12 items to assess thumb-specific symptoms and disability. Scoring for this measure is identical to that of the quick DASH. Radiographic severity for each patient was assessed by a blinded reviewer using the Eaton-Littler classification system (29). Painful joint count was collected using a homunculus form and has been previously used in studies of osteoarthritis (30–33). In brief: total joint count was derived from homunculus data and represented a value out of a maximum 28 points. The neck, upper back, mid back, lower back, shoulders, elbows, wrists, hips, knees, ankles and mid-feet were valued at 1 point when marked. In each hand, metacarpophalangeal joints, proximal interphalangeal joints, and distal interphalangeal joints were grouped and valued at 1 point if one or more of those joints were marked (for maximum of 3 points per hand). In each foot, metatarsophalangeal joints and interphalangeal joints were grouped and valued at 1 point if one or more of those joints were marked (for a maximum of 2 points per foot).

### Cytokine Quantification

Expression of inflammatory cytokines was measured using the Bio-rad Bio-Plex Pro Human Cytokine 27-Plex Assay kit, read on a Luminex 200 system and analyzed using Luminex xPotent Software as per product specifications. The evaluated cytokines consisted of: basic fibroblast growth factor (bFGF), eotaxin, granulocyte colony-stimulating factor (G-CSF), granulocyte macrophage colony-stimulating factor (GM-CSF), interferon gamma (IFNg), interleukin-1 receptor antagonist (IL-1RA), Interleukins (IL-1b, IL-2, IL-4, IL-5, IL-6, IL-7, IL-8, IL-9, IL-10, IL-12p70, IL-13, IL-15, IL-17a, interferon gamma inducible protein 10 (IP-10), monocyte chemoattractant protein 1 (MCP-1), macrophage inflammatory protein (MIP-1a, MIP-1b), platelet derived growth factor bb (PDGF-bb), regulated on activation normal T-cell expressed and secreted, tumor necrosis factor alpha (TNFa), vascular endothelial growth factor (VEGF). Plasma samples were measured at baseline (pre-treatment), from either non-surgically managed (n=44) or surgical (n=39) patients, or post-surgically at 6-weeks (n=31), 3 months (n=24), 6 months (n=22) or 1 year (n=17). Samples from the different groups and time-points were randomly allocated to plates, and the experiment was conducted using de-identified samples run in duplicate. Cytokine concentration was calculated based on standard curve as per product manual.

### Statistical Analyses

Statistical analyses were performed using R version 3.6.2. Clinical variables (age, sex, BMI, quick DASH, TASD, TASD- subscales symptoms and disability, key pinch strength, grip strength, and painful joint count) were analyzed using bivariate methods (**Table 1**). Cytokine concentrations were log (x+1) transformed, mean-centered and scaled by their respective standard deviation in order to mitigate the influence of extreme values and to facilitate interpretation of model coefficients. Principal Component Analysis (PCA) was performed on transformed cytokine data, and used for data visualization and cluster generation (34). Cytokines which had greater than 70% of values below the lower limit of detection were excluded. Wilcoxon tests were performed to examine differences in cytokine levels between treatment groups, sexes, and clusters. Adjusted associations between each cytokine at baseline and each clinical outcome at baseline (adjusting for age, sex, BMI and painful joint count) were assessed using Generalized Linear Models. Additionally, associations between baseline cytokines and change scores in the outcomes (baseline vs 6 months and baseline vs 12 months), as well as associations between change scores in the cytokines (baseline vs 6 months and baseline vs 12 months) and outcomes (at 6 and 12 months respectively), were similarly assessed (using GLM framework). P-values were adjusted using the method of Benjamin and Hochberg to maintain a false discovery rate of 0.1 (35).

**Table 1:** Baseline Patient Characteristics.

		Non-Surgical Management	Surgery	p-value
n		44	39	
Age [mean (SD)]		62.80 (10.24)	60.07 (8.11)	0.185
Sex (%)	FEMALE	31 (70.5)	27 (69.2)	1
	MALE	13 (29.5)	12 (30.8)	
BMI [median (IQR)]		25.18 [22.86, 28.27]	28.29 [24.38, 32.86]	0.01
Quick DASH [mean (SD)]		35.52 (18.20)	55.54 (18.93)	<0.001
VAS [median (IQR)]		75.50 [61.00, 90.00]	76.00 [70.00, 85.00]	0.913
TASD [mean (SD)]		42.28 (17.81)	61.27 (17.18)	**<0.001**
TASD Subscale Symptom [mean (SD)]		41.72 (18.64)	62.27 (16.16)	**<0.001**
TASD Subscale Disability [median (IQR)]		40.00 [30.00, 56.25]	60.00 [50.00, 75.00]	**<0.001**
XRAY grade (%)	1	0 (0.0)	1 (2.9)	0.103
	2	9 (22.0)	11 (31.4)	
	3	22 (53.7)	10 (28.6)	
	4	10 (24.4)	13 (37.1)	
Key Pinch Strength [mean (SD)]		5.48 (2.43)	4.80 (2.32)	0.208
Maximum Grip Strength [mean (SD)]		25.09 (10.66)	21.42 (11.40)	0.136
Total Joint Count [median (IQR)]		9.00 [5.00, 13.00]	8.00 [4.00, 12.00]	0.731

BMI, Body Mass Index; QDASH, quick Disability of Arm; Shoulder and Hand score; VAS, Visual Analogue Scale; TASD, Trapeziometacarpal Arthrosis Symptom and Disability Questionnaire; X-Ray Grade, Eaton- Littler classification 1-4 (most severe); Joint Count, painful joints as indicated by patient homunculus. Bold values indicate statistically significant values.

## Results

### Patients Undergoing Surgery Have More Disability, Higher Pain and BMI

Patient anthropometric data, reported outcome measures, and functional assessments are reported in **Table 1**. There were no significant differences in age or sex between the surgical and non-surgically managed group. Approximately 70% of the study population was female, nearly evenly distributed between surgical and non-surgical treatment modalities. No statistical differences were detected in VAS patient reported pain, radiographic disease severity, key pinch strength, grip strength or total number of painful joints between groups.

Patients undergoing surgery had significantly higher BMI than those undertaking non-surgical treatment (28.29 [24.38,32.86] vs 25.18 [22.86, 28.27], p=0.010). The surgical group also reported more severe pain and disability, as indicated by quick DASH scores (55.54± 18.93 vs 35.52± 18.20, p<0.001), and TASD total scores (61.27± 17.18 vs 42.28 ± 17.81, p<0.001), which was reflected in both of the TASD sub-scales of symptoms (62.27±16.16 vs 41.72±18.64, p<0.001), and disability (60.00 [50.00, 70.00] vs 40.00 [30.00, 56.25], p<0.001).

### IL-7 Can Discriminate Between Surgical and Non-Surgical Patients

**Figure 1:**
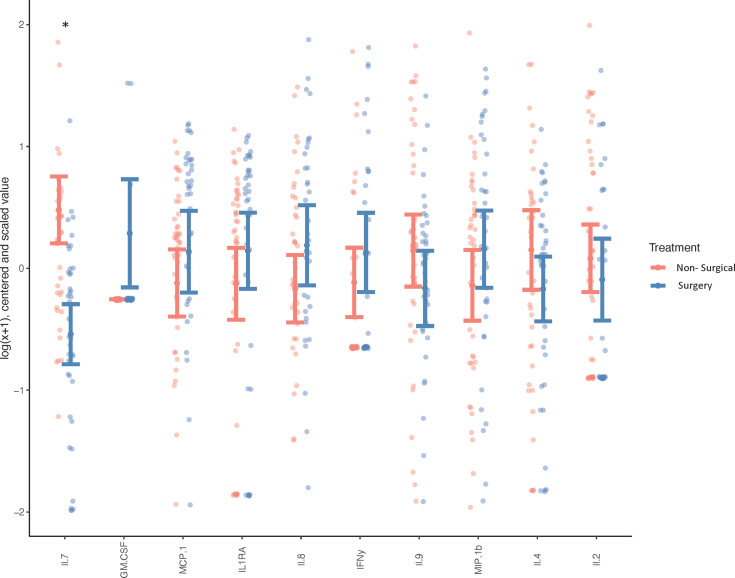
Systemic Cytokine Expression in Surgical vs Non-surgically Managed Patients. Differences in circulating cytokines in the plasma of non-surgically managed (n=44) or surgically managed (n=39) patients at baseline. Wilcoxon test, *p < 0.0001, q < 0.0001, less than 5% of values below lower limit of detection. Adjusted for age, sex, BMI and painful joint count, the y-axis depicts cytokine concentrations (which were log[x+1] transformed, mean-centred and scaled by their respective standard deviation, while cytokines are labelled on the x-axis.

Biochemical profiling using targeted panel cytokine screening was used to examine whether there were systemic differences in the plasma of surgical compared to non-surgically managed patients (**Figure 1**). The levels of IL-7 (q<0.00001) were significantly different between the two groups. Patients with higher relative levels of IL-7 had a decreased likelihood of going into surgery with an adjusted odds ratio of 0.220 (q<0.05) (**Table 2**). None of the other cytokines tested were effective at discriminating surgical status. Data for cytokine screening in pg/ml, as well as baseline demographic and clinical measures can be accessed in **Supplementary Table 6**.

**Table 2:** Cytokine Odds Ratio For Surgical vs. Non-surgical Management.

Cytokine	Adjusted Odds Ratio	lower 0.025	upper 0.975	p-value	q-value
IL-7	0.22	0.084	0.464	0.00044	**0.011433**
Eotaxin	1.636	0.982	2.865	0.06851	0.596163
MCP-1	1.54	0.948	2.616	0.090862	0.596163
IL-8	1.55	0.949	2.664	0.091717	0.596163
MIP-1a	1.441	0.88	2.43	0.153451	0.712417
MIP-1b	1.371	0.853	2.269	0.201402	0.712417

Adjusted (age, sex, BMI, painful joint count) odds ratio describing likelihood of undergoing surgical treatment with increased levels of cytokine expression in non-surgically managed (n=44) or surgically managed (n=39) patients at baseline.

Bold values indicate statistically significant values.

As females comprised more than two-thirds of the study population, we investigated whether there were differences in biochemical profiles between sexes. There were no differences in cytokine expression between sexes after correcting for false discovery rate (**Supplementary Figure 2**). Associations between radiographic severity, patient reported outcome measures, functional assays and cytokine expression in surgical and non-surgically managed patients were evaluated. After adjusting for clinical factors (age, sex, BMI, painful joint count), there were few significant associations between functional tests and cytokine expression but not among other measures (**Supplementary Tables 1–4**).

### Identification of a Unique Molecular Signature in TMOA Patients

**Figure 2:**
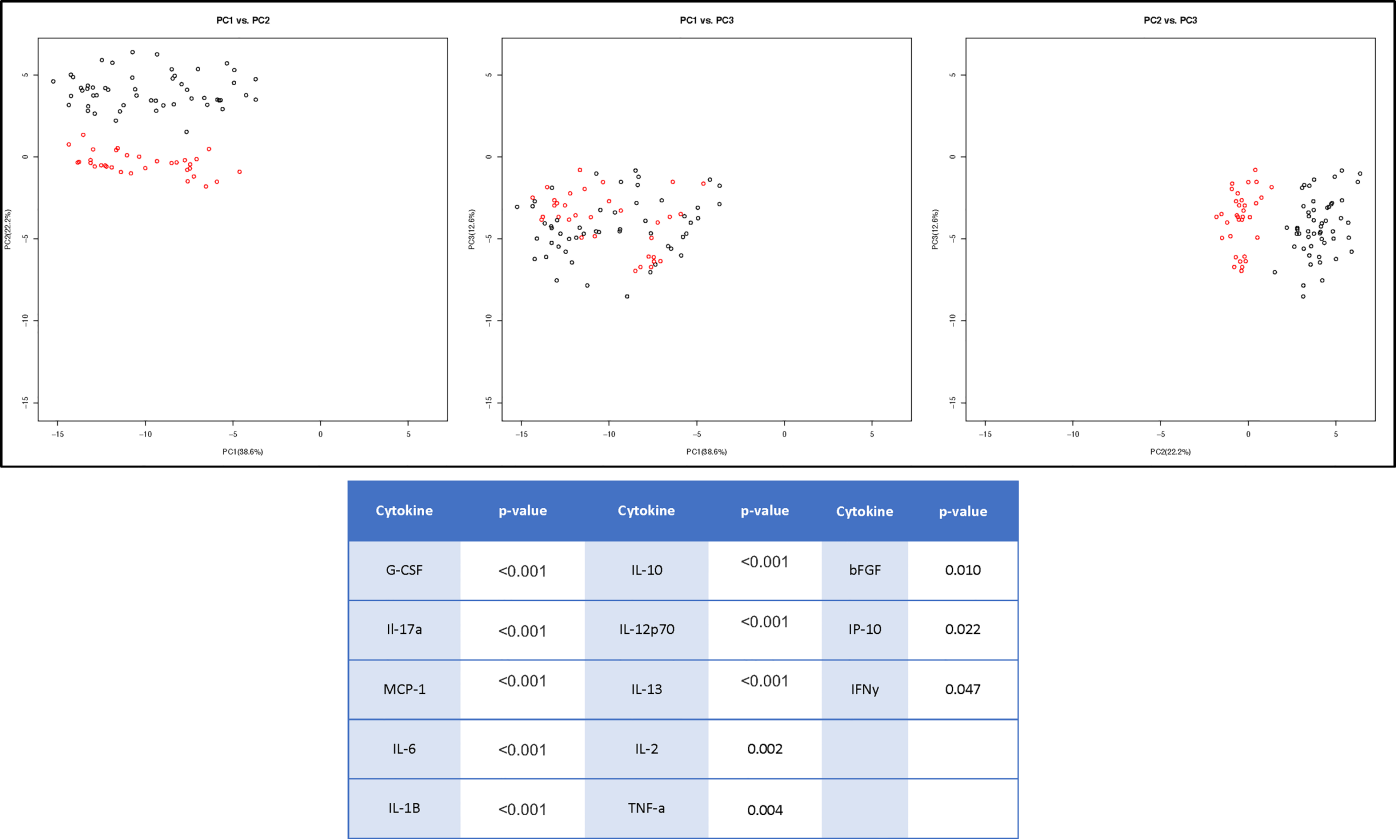
Cluster Analysis of TMOA Patients Indicates Presence of Sub-Groups. Principal component analysis (n=83) of cytokine expression of patients at baseline timepoint indicates a two sub-groups of patients. Wilcoxon tests performed between the two clusters indicates 13 cytokines which are differentially expressed (p < 0.05, q < 0.1).

**Figure 3:**
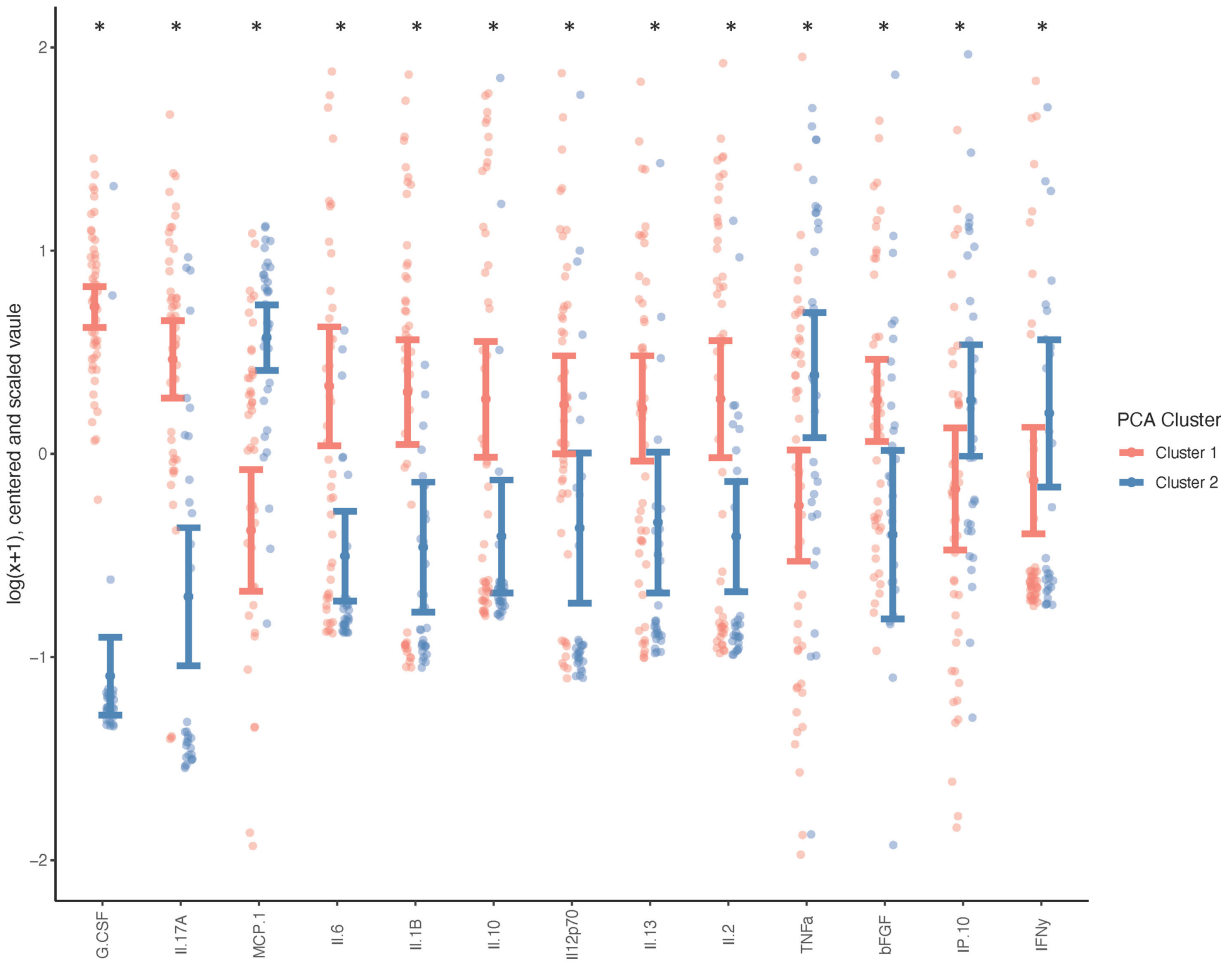
Systemic Cytokine Differences between Patient Clusters. Wilcoxon test was performed on cytokines indicated as differently expressed between clusters (n=50 cluster 1, n=33 cluster 2), and adjusted for age, sex, BMI and total joint count (*p < 0.05, q < 0.1) and graph indicates the direction of differential expression between the two clusters.

Due to the minimal associations found between cytokine expression and clinical outcome measures, we sought to determine whether there were endogenous differences between patients that could explain the heterogeneity seen within surgical and non-surgical patient groups. Using Principal Component Analysis (PCA), we conducted an unbiased examination of cytokine expression in all patients and observed that patients segregated into two clusters, regardless of surgical status, age, sex, joint count or BMI (**Figure 2**). Two patient clusters emerged based on a unique molecular signature consisting of 13 cytokines: G-CSF, IL-17a, MCP-1, IL-6, IL-1b, IL-10, IL-12p70, Il-13, IL-2, TNFa, B-FGF, IP-10 and IFNγ. (q<0.02). The difference in each of these cytokines between the two clusters is visualized in **Figure 3**, where relative increase or decrease is seen in the latter. A summary of these cytokines and brief examination of their role within the context of OA can be found in **Supplementary Table 5**.

## Discussion

Currently there are no validated biomarkers of TMOA, and as such, there are limited options available to patients during early-stage disease when prognosis is largely unknown. A plasma biomarker, which is able to distinguish disease severity, is a feasible, minimally invasive option that could enable clinicians to make prognosis driven treatment recommendations as more efficacious therapies become available. In order to be able to identify biomarkers for TMOA, characterizing molecular profiles of the disease are required to foster an understanding of the complex regulatory environment. In this study, we used cytokine multiplex assays paired with matched patient clinical information to show that IL-7 is a molecular indicator, which could potentially differentiate between patients who are stably non-surgically managed, and those with advanced disease needing surgery. We also discovered that patients with TMOA can self-segregate into groups that are defined by a specific molecular signature indicating that phenotypes related to biological differences in TMOA may exist.

In order to create a comprehensive clinical picture of our study population, we compared patients undergoing non-surgical management to those undergoing trapeziectomy +\- tendon interposition. We found that although demographic and clinical measures were similarly distributed between groups, BMI and patient reported pain and function scores (TASD, quick DASH) were significantly different. Few studies have compared surgical and non-surgical management directly, though in these studies similar clinical characteristics (such as age and radiographic disease severity) are observed between the two patient groups (36, 37). These observations support literature describing radiographic severity and pain (the primary indication for surgery) as discordant. In fact, the decision to perform surgery for TMOA has been reported as largely subjective (38). Additionally, psychological factors such as illness perception and pain catastrophizing account for 42% of patient pre-treatment pain levels, whereas patient characteristics including radiographic disease severity only accounts for only 6%, leaving more than half of pain levels to unknown contributing factors (39). Biochemical factors such as cytokines may also be an important contributing factor that could be objectively measured allowing for comparisons within and between patients.

Cytokines that are able to distinguish disease severity could aid in defining mechanisms and finding molecular indicators capable of diagnosis and prognosis. Our adjusted analysis demonstrated that circulating IL-7 in plasma can distinguish between patients undergoing surgical or non-surgical management, despite similar radiographic disease severity, hand function measures and number of painful joints. This indicates that systemic levels of IL-7 could distinguish symptomatic disease severity in individuals with OA in multiple joints. Further, increased circulating IL-7 is associated with a significant decrease in likelihood of being symptomatic and requiring surgery (OR=0.220). Overall, this indicates the need to further study circulating IL-7 in TMOA patient populations in order to determine if it could be used as a blood-based biomarker to monitor patients at risk for TMOA progression, to intervene with more aggressive treatment or preventative measures as they become available, or to plan for surgery. It is important to note that though our study accounted for the number of painful joints as a proxy for symptomatic OA in multiple joints, it does not account for the severity of OA in these joints which could be a significant contributing factor to circulating cytokine levels.

IL-7 is a growth factor involved in the development of B and T cells, but is also produced by variety of cell types including chondrocytes (20, 40). In a Han Chinese population, polymorphisms in the IL-7 gene are associated with increased risk of developing OA (41). In knee OA, IL-7 in synovial fluid was demonstrated to correlate positively with patient age, but was depressed in patients with severe osteoarthritis affecting multiple knee compartments (42). Increased local levels of IL-7 are typically considered pro-inflammatory and part of the senescence associated secretory phenotype (43, 44). *In-vitro*, IL-7 works to promote articular cartilage destruction through the up-regulation of cartilage destructive protease MMP-13 (40). Recent data from the Osteoarthritis Initiative cohort also indicates that significantly decreased levels of IL-7 are present in accelerated hand OA which progresses from minimal radiographic disease to end-stage over the course of 48 months (accepted abstract) (45). If indeed, IL-7 could be validated as a prognostic biomarker for TMOA then it may help to identify individuals who progress through this condition at different rates. Prognostic biomarkers can also be useful in facilitating understanding of disease pathogenesis, differentiating phenotypes within a heterogenous OA population, and comparing treatment outcomes during clinical trials where imaging outcomes may not reflect active disease (46). It is important to note none of the other 26 cytokines evaluated were capable of this measure after adjusting for painful joint count, age, sex and BMI, indicating the specificity of IL-7 as a molecular indicator of TMOA disease severity even when measured systemically.

Associations (MIP1a, MIP1b, Il-2 and bFGF) were observed in the relationship between the change in cytokine expression over time and some clinical outcome measures after adjusting for age, sex, BMI and painful joint count. Though these relationships were not consistently statistically significant across time points or outcome measures, some understanding may be drawn from current literature. MIP1a (CCL3) has previously been suggested as an early predictor of surgical outcome in non-union fracture therapy, implicated in likelihood of total-knee arthroplasty revisions, and as part of the senescence-associated secretory phenotype prediction of adverse post-surgical outcomes (47, 48). Both CCL3 and CCL4 (MIP1a, MIP1b) are elevated in revision total knee arthroplasty (TKA) patients compared to primary TKA patients, indicating their potential as predictors for post-surgical outcome (49). Whereas elevation of synovial fluid FGF2 (bFGF) reflects clinical response after joint distraction (50). However, within the literature the differences among source tissue and biofluids, as well as types and timing of measurements make it difficult to draw conclusions.

The heterogeneity in outcome measures within our non-surgically and surgically managed patient groups and the potential for treatment decisions to be influenced by patient and surgeon bias, prompted us to perform an unbiased examination of cytokine expression through Principal Component Analysis. It was observed that the data self-segregated into two visually discernable groups which differed based on the expression of unique combination of 13 cytokines; a molecular signature. This molecular signature consists of G-CSF, IL-17a, IL-6, IL-10, IL-1b, IL-12p70, IL-2, IL-13, MCP-1, B-FGF, IP-10, TNFa, and IFNy. In Cluster 1 G-CSF, IL-17a, IL-1b, IL-6, IL-10, IL-12p70, IL-13, IL-2 and B-FGF are elevated whereas in Cluster 2 MCP-1,TNFa, IP-10 and IFNy are elevated. These clusters cannot be attributed to difference in age, BMI, surgical status, patient reported outcome measures, or functional performance. The divergence of patients based solely on differing expression profiles of cytokines suggests that these subgroups may be indicative of endogenous phenotypes. The observed heterogeneity can also help inform that pharmacological therapies, prognostic, or diagnostic tools may have to be targeted to specific subpopulations to be successful.

Recent OA research has focused on understanding molecular and clinical phenotypes of this disease, of which there may be many (51). Common clinical phenotypes include chronic pain, mechanical overload, metabolic syndrome, bone and cartilage metabolism, minimal joint disease, and inflammatory phenotypes, while the senescence associated secretory phenotype is a distinct and well characterized molecular endotype (52). To our knowledge, this is the first study to show different molecular phenotypes in TMOA. The implication of an inflammation driven phenotype may signify that a subset of these patients may be more responsive to therapies precisely targeting these pathways, and understanding these pathological mechanisms will be crucial to developing effective therapies.

Limitations of the current study include a lack of a healthy comparator group, and decreased patient numbers during follow-up time points which may have impacted our ability to detect post-surgical differences in cytokine expression. The lack of a healthy patient group could indicate that the endogenous phenotypes we see within the TMOA population, could be true of the general population or other specific populations. Investigation of a healthy cohort would yield a valuable comparison to our molecular clusters, as well as surgical and non-surgical groups. In the current study we have not investigated a healthy control group due to the substantial (decades) age difference between the groups. Inclusion of a healthy control group could influence the interpretation of results by permitting the evaluation of the dispersion of circulating IL-7 levels between healthy, stably managed non-surgical as well as surgical patients. Hypothetically, if IL-7 levels were observed as similar between healthy controls and non-surgical TMOA patients, this could indicate that the relative decrease in IL-7 levels may be associated with mechanisms or pathways involved in more symptomatic TMOA. However, if levels of IL-7 in a healthy comparator were similar to those in the surgical group (or in-between groups), it could indicate that relatively different levels between groups is the result of a factor external to those considered within the current study.

One source of bias in our study, may be the patient’s decision to have surgery which could be based on a myriad of factors including their perception of surgery, convenience, time off work, and external support among others. Additionally, more precise imaging methods or analysis techniques such as quantifying osteophyte size, synovitis with ultrasound or MRI may more closely relate pain to structural damage than gross radiographic scores (53). In addition, though we present IL-7 as a cytokine capable of distinguishing between surgical and non-surgical TMOA patients, it should be noted that systemic cytokines are reflective of multiple active processes occurring concurrently throughout the body and not necessarily attributable to a specific process in a specific joint. It is also considered that the correlation between increased levels of this cytokine to decreased symptoms and likelihood of surgery may be an association and may not be due to causation, such as having a physiological role mediating this disease. Rather, it is possible that the association between these variables could be explained by factors which were not measured in the current study. Establishing a causative physiological role for the association observed would require additional interventional or experimental studies. Lastly, the cytokine expression and unique molecular signature discovered in this study will need to be validated in external cohorts to determine whether these results are applicable and reproducible in other TMOA populations.

TMOA, when symptomatic, has serious implications for hand function impacting vocational and avocational activities. Systemic IL-7 can distinguish between patients with disease severe enough to undergo surgery and less symptomatic patients. Elevated levels of IL-7 is associated with decreased likelihood to undergo surgery. This observation, if validated in other populations, could have the potential to provide an effective objective method to monitor patient response to non-surgical intervention. Interestingly, regardless of clinical variables, TMOA patients also segregate into two sub-groups based on the expression of a combination of 13 cytokines indicating that there may be endogenous phenotypes in TMOA that could be precisely targeted for more effective treatment.

## Data Availability Statement

The raw data supporting the conclusions of this article will be made available by the authors, without undue reservation.

## Ethics Statement

The studies involving human participants were reviewed and approved by University Health Network Research Ethics Board. The patients/participants provided their written informed consent to participate in this study.

## Author Contributions

AR, JR, MK, and HB contributed to conception and design of the study. DA was responsible for clinical data collection. KS and JM performed the statistical analysis. AR wrote the the manuscript. All authors contributed to manuscript revision, read, and approved the submitted version.

## Funding

This study was supported in part by grants to HB by the American Foundation for Surgery of the Hand Clinical Grant (#1407) and the Education Foundation of the Canadian Society of Plastic Surgeons. MK was supported in part by funding from the Canada Research Chairs Program (#950-232237), Canada Foundation for Innovation (#35171), Tony and Shari Fell Platinum Chair in Arthritis Research and Schroeder Arthritis Institute *via* the University Health Network Foundation (Toronto). AR is the recipient of the Canadian Institute of Health Research Postdoctoral Fellowship Award and the Krembil Research Institute Postdoctoral Fellowship Award.

## Conflict of Interest

The authors declare that the research was conducted in the absence of any commercial or financial relationships that could be construed as a potential conflict of interest.

## Publisher’s Note

All claims expressed in this article are solely those of the authors and do not necessarily represent those of their affiliated organizations, or those of the publisher, the editors and the reviewers. Any product that may be evaluated in this article, or claim that may be made by its manufacturer, is not guaranteed or endorsed by the publisher.

## Supplementary Material

Supplementary Figure 1 Patient Pipeline. Patients followed the pipeline in the schematic above. In brief: patients undergoing non-surgical or surgical treatment for trapeziometacarpal osteoarthritis were recruited to the study and followed for 52 weeks. Patient reported outcome measures (PROM), key pinch strength and grip strength (clinical tests), as well as blood, urine and tissues (surgical group only) were collected.

Supplementary Figure 2 Differences in Cytokine Expression Between Sexes in TMOA Patients.There were no differences in systemic cytokine expression between male or female patients at baseline after correcting for false discovery rate. (n=58 females: 31 nonsurgical/ 28 surgical, 25 males: 13 non-surgical/ 12 surgical, Wilcoxon Test q > 0.1).

Supplementary Table 1 Radiographic Scores do not correlate with systemic cytokine expression. After adjusting for age, sex, BMI, and painful joint count, there were no significant associations between radiographic severity as determined by Eaton-Littler scores and systemic cytokine expression in surgical or non-surgical patient groups at baseline (n=44 non-surgical,39 surgical, Wilcoxon Test, q > 0.1).

Supplementary Table 2 PROM are not associated with systemic cytokine expression. After adjusting for age, sex, BMI, and painful joint count, there were no significant associations between Patient Reported Outcome Measures and systemic cytokine expression in surgical or non-surgical patient groups at baseline (n=44 non-surgical, 39 surgical, Wilcoxon Test, q > 0.1), in either the quick DASH, VAS, or TASD (total, symptomatic or disability scores).

Supplementary Table 3 Clinical Function is not associated with systemic cytokine expression. After adjusting for age, sex, BMI, and painful joint count, there were no significant associations between clinical function and systemic cytokine expression in surgical or non-surgical patient groups at baseline (n=44 non-surgical, 39 surgical, Wilcoxon Test, q > 0.1), in key pinch strength.

Supplementary Table 4 Associations between clinical outcomes and cytokine expression over time. After adjusting for age, sex, BMI and painful joint count there were few significant associations between change in cytokine expression over time and clinical outcome, (n= 44 surgical baseline, n=20-22 6 months, n=16-17 one-year, *p < 0.05, q < 0.1, bolded). BL-6M and BL-1Y describe the change in expression from Baseline to 6 Months, or Baseline to 1 Year.

Supplementary Table 5 Roles and OA context of cytokines which differentiate patient subgroups. The traditional role of each cytokine or chemokine, as well as well as its associations within OA literature are described.

Supplementary Table 6 Baseline Data. Baseline data for surgical and non-surgical patients, including clinical characteristics, PROM, functional scores and baseline cytokine measurements (pg/ml) are reported.
