# Supplementary figures and images for: Investigating Molecular Signatures Underlying Trapeziometacarpal Osteoarthritis Through the Evaluation of Systemic Cytokine Expression
Source: Front Immunol. 2022 Jan 20;12:794792. doi: 10.3389/fimmu.2021.794792 (PMC8814933; doi:10.3389/fimmu.2021.794792)

Supplementary Figure 1

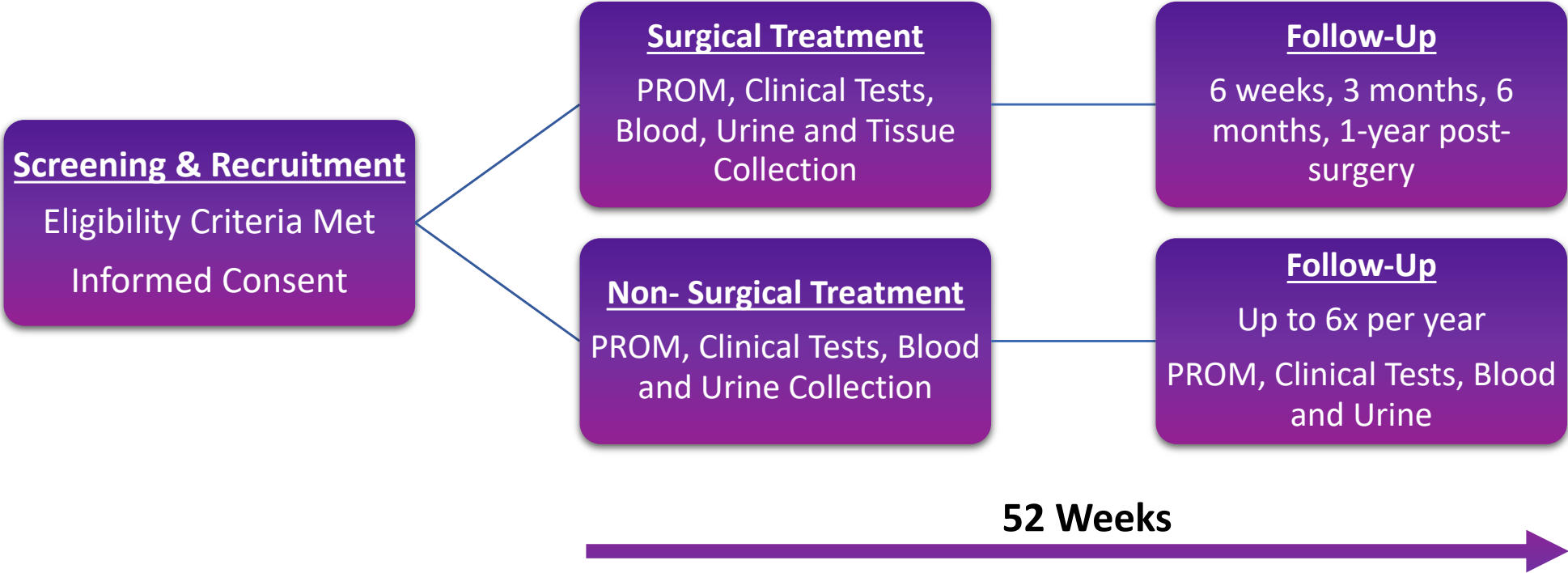

Supplement: Supplementary Figure 1 — Patient Pipeline. Patients followed the pipeline in the schematic above. In brief: patients undergoing non-surgical or surgical treatment for trapeziometacarpal osteoarthritis were recruited to the study and followed for 52 weeks. Patient reported outcome measures (PROM), key pinch strength and grip strength (clinical tests), as well as blood, urine and tissues (surgical group only) were collected. [file Image_1.pdf]

Supplementary Figure 2

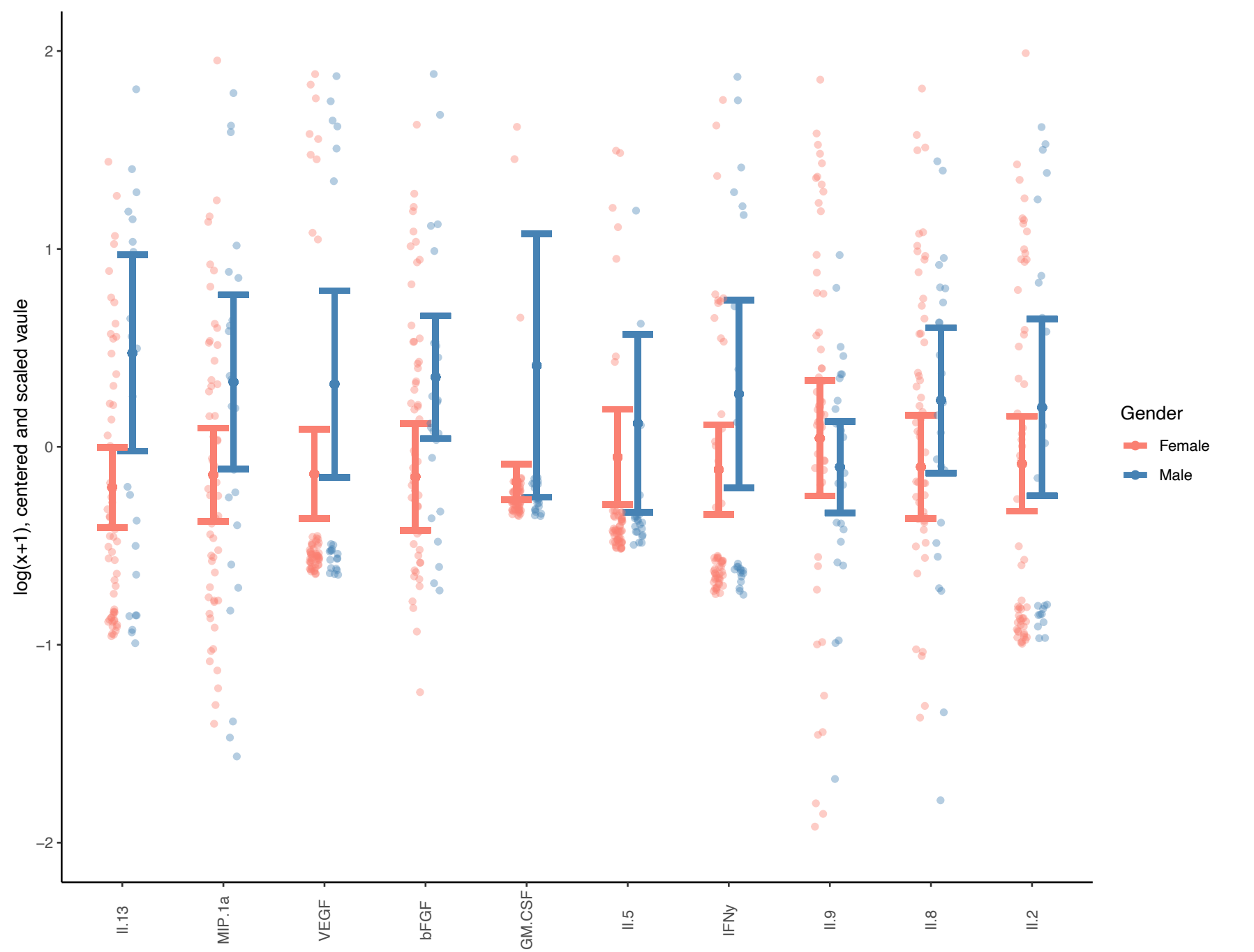

Supplement: Supplementary Figure 2 — Differences in Cytokine Expression Between Sexes in TMOA Patients.There were no differences in systemic cytokine expression between male or female patients at baseline after correcting for false discovery rate. (n=58 females: 31 nonsurgical/ 28 surgical, 25 males: 13 non-surgical/ 12 surgical, Wilcoxon Test q > 0.1). [file Image_2.pdf]
