# Supplementary material for: Investigating Molecular Signatures Underlying Trapeziometacarpal Osteoarthritis Through the Evaluation of Systemic Cytokine Expression
Source: Front Immunol. 2022 Jan 20;12:794792. doi: 10.3389/fimmu.2021.794792 (PMC8814933; doi:10.3389/fimmu.2021.794792)
Supplement: Supplementary Table 1 — Radiographic Scores do not correlate with systemic cytokine expression. After adjusting for age, sex, BMI, and painful joint count, there were no significant associations between radiographic severity as determined by Eaton-Littler scores and systemic cytokine expression in surgical or non-surgical patient groups at baseline (n=44 non-surgical,39 surgical, Wilcoxon Test, q > 0.1). [file Table_1.docx]

**Supplementary Table 1:**

|  |  |  |  |  |  |  |
| --- | --- | --- | --- | --- | --- | --- |
| **Category** | **Cytokine** | **Estimate** | **Lower 0.025** | **Upper 0.025** | **p-value** | **q-value** |
| X-Ray Grade | GM-CSF | 0.56 | 0.326 | 0.893 | 0.028292254 | 0.735598606 |
|  | Il-1B | 0.623 | 0.394 | 0.971 | 0.073371047 | 0.942366106 |
|  | Il-10 | 0.7 | 0.453 | 1.072 | 0.177227388 | 0.942366106 |
|  | PDGF-BB | 0.7 | 0.447 | 1.081 | 0.177315075 | 0.942366106 |
|  | Il-7 | 1.407 | 0.87 | 2.315 | 0.19652471 | 0.942366106 |
|  | Il-8 | 0.722 | 0.44 | 1.162 | 0.217469101 | 0.942366106 |
