# Supplementary material for: Investigating Molecular Signatures Underlying Trapeziometacarpal Osteoarthritis Through the Evaluation of Systemic Cytokine Expression
Source: Front Immunol. 2022 Jan 20;12:794792. doi: 10.3389/fimmu.2021.794792 (PMC8814933; doi:10.3389/fimmu.2021.794792)
Supplement: Supplementary Table 2 — PROM are not associated with systemic cytokine expression. After adjusting for age, sex, BMI, and painful joint count, there were no significant associations between Patient Reported Outcome Measures and systemic cytokine expression in surgical or non-surgical patient groups at baseline (n=44 non-surgical, 39 surgical, Wilcoxon Test, q > 0.1), in either the quick DASH, VAS, or TASD (total, symptomatic or disability scores). [file Table_2.docx]

| **Supplementary Table 2:** | |  |  |  |  |  |
| --- | --- | --- | --- | --- | --- | --- |
|  |  |  |  |  |  |  |
| **Category** | **Cytokine** | **Estimate** | **Lower 0.025** | **Upper 0.025** | **p-value** | **q-value** |
| quick DASH Score | Il-17A | 6.25 | 1.927 | 10.573 | 0.005180539 | 0.134694023 |
|  | Il-10 | 4.689 | 0.323 | 9.055 | 0.035666024 | 0.3189389 |
|  | Il-13 | 4.971 | 0.313 | 9.629 | 0.036800642 | 0.3189389 |
|  | Il-1B | 4.36 | -0.055 | 8.775 | 0.052863212 | 0.343610878 |
|  | Il-2 | 4.089 | -0.487 | 8.664 | 0.079081806 | 0.356671804 |
|  | G-CSF | 3.868 | -0.634 | 8.369 | 0.091088836 | 0.356671804 |
|  |  |  |  |  |  |  |
| VAS Score | Il-12p70 | -5.521 | -9.94 | -1.102 | 0.015050274 | 0.227957123 |
|  | G-CSF | -4.975 | -9.194 | -0.756 | 0.021486884 | 0.227957123 |
|  | Il-6 | -5.042 | -9.49 | -0.594 | 0.026859282 | 0.227957123 |
|  | IP-10 | 5.166 | 0.372 | 9.961 | 0.035070327 | 0.227957123 |
|  | Il-13 | -4.201 | -8.667 | 0.265 | 0.064801585 | 0.29137552 |
|  | Il-17A | -3.979 | -8.247 | 0.29 | 0.067240505 | 0.29137552 |
|  |  |  |  |  |  |  |
| TASD Overall Score | Il-17A | 4.659 | 0.607 | 8.712 | 0.024820785 | 0.376389508 |
|  | Il-10 | 4.239 | 0.217 | 8.26 | 0.039128068 | 0.376389508 |
|  | Eotaxin | 4.151 | -0.111 | 8.412 | 0.056114438 | 0.376389508 |
|  | G-CSF | 3.877 | -0.25 | 8.003 | 0.065164364 | 0.376389508 |
|  | Il-1B | 3.708 | -0.37 | 7.786 | 0.074089588 | 0.376389508 |
|  | Il-9 | -3.576 | -7.682 | 0.53 | 0.086859117 | 0.376389508 |
|  |  |  |  |  |  |  |
| TASD Symptom Score | Eotaxin | 5.34 | 1.157 | 9.524 | 0.013049755 | 0.241984781 |
|  | Il-17A | 4.624 | 0.576 | 8.672 | 0.025718447 | 0.241984781 |
|  | Il-10 | 4.501 | 0.502 | 8.501 | 0.027921321 | 0.241984781 |
|  | Il-9 | -3.864 | -7.949 | 0.221 | 0.063411191 | 0.311751182 |
|  | IL-1B | 3.796 | -0.271 | 7.863 | 0.066880502 | 0.311751182 |
|  | G-CSF | 3.779 | -0.345 | 7.903 | 0.07194258 | 0.311751182 |
|  |  |  |  |  |  |  |
| TASD Subscale Disability | IL-17A | 4.709 | -0.12 | 9.537 | 0.055801175 | 0.650035405 |
|  | G-CSF | 4.014 | -0.883 | 8.911 | 0.1066688 | 0.650035405 |
|  | Il-10 | 3.871 | -0.931 | 8.674 | 0.112530691 | 0.650035405 |
|  | Il-1B | 3.585 | -1.263 | 8.433 | 0.144927149 | 0.650035405 |
|  | Il-13 | 3.503 | -1.644 | 8.649 | 0.179221712 | 0.650035405 |
|  | RANTES | -3.278 | -8.14 | 1.584 | 0.183340993 | 0.650035405 |
