# Supplementary material for: Investigating Molecular Signatures Underlying Trapeziometacarpal Osteoarthritis Through the Evaluation of Systemic Cytokine Expression
Source: Front Immunol. 2022 Jan 20;12:794792. doi: 10.3389/fimmu.2021.794792 (PMC8814933; doi:10.3389/fimmu.2021.794792)
Supplement: Supplementary Table 3 — Clinical Function is not associated with systemic cytokine expression. After adjusting for age, sex, BMI, and painful joint count, there were no significant associations between clinical function and systemic cytokine expression in surgical or non-surgical patient groups at baseline (n=44 non-surgical, 39 surgical, Wilcoxon Test, q > 0.1), in key pinch strength. [file Table_3.docx]

| **Supplementary Table 3:** | |  |  |  |  |  |
| --- | --- | --- | --- | --- | --- | --- |
|  |  |  |  |  |  |  |
|  |  |  |  |  |  |  |
| **Category** | **Cytokine** | **Estimate** | **Lower 0.025** | **Upper 0.025** | **p-value** | **q-value** |
| Key Pinch Strength | G-CSF | -0.432 | -0.794 | -0.069 | 0.020210092 | 0.525462389 |
|  | Il-6 | -0.391 | -0.772 | -0.009 | 0.04495324 | 0.564101444 |
|  | Il-17A | -0.345 | -0.712 | 0.022 | 0.065088628 | 0.564101444 |
|  | Il-1B | -0.256 | -0.625 | 0.112 | 0.169882144 | 0.820417288 |
|  | Il-1RA | 0.236 | -0.149 | 0.622 | 0.225935096 | 0.820417288 |
|  | Il-7 | 0.213 | -0.178 | 0.605 | 0.280961176 | 0.820417288 |
