# Supplementary material for: Investigating Molecular Signatures Underlying Trapeziometacarpal Osteoarthritis Through the Evaluation of Systemic Cytokine Expression
Source: Front Immunol. 2022 Jan 20;12:794792. doi: 10.3389/fimmu.2021.794792 (PMC8814933; doi:10.3389/fimmu.2021.794792)
Supplement: Supplementary Table 4 — Associations between clinical outcomes and cytokine expression over time. After adjusting for age, sex, BMI and painful joint count there were few significant associations between change in cytokine expression over time and clinical outcome, (n= 44 surgical baseline, n=20-22 6 months, n=16-17 one-year, *p < 0.05, q < 0.1, bolded). BL-6M and BL-1Y describe the change in expression from Baseline to 6 Months, or Baseline to 1 Year. [file Table_4.docx]

**Supplementary Table 4**

|  |  |  |  |  |  |  |  |
| --- | --- | --- | --- | --- | --- | --- | --- |
| **Category** | **n** | **Cytokine** | **Estimate** | **Lower 0.025** | **Upper 0.025** | **p-value** | **q-value** |
| Key Pinch BL-6M | 22 | MIP-1b | 0.025 | 0.006 | 0.044 | 0.012409039 | 0.33504406 |
|  | 21 | IL-17A | 0.177 | 0.005 | 0.349 | 0.044540588 | 0.4993301 |
|  | 22 | MIP-1a | 1.005 | -0.027 | 2.036 | 0.055481122 | 0.4993301 |
|  | 22 | PDGF-BB | 0.002 | 0 | 0.004 | 0.093075764 | 0.5881243 |
|  | 22 | bFGF | 0.046 | -0.016 | 0.108 | 0.133439514 | 0.5881243 |
|  | 22 | IL-1RA | 0.006 | -0.002 | 0.014 | 0.138237789 | 0.5881243 |
| Key Pinch BL-1Y | 17 | bFGF | 0.084 | 0.035 | 0.132 | 0.002636904 | **0.06016334** |
|  | 17 | IL-2 | 0.662 | 0.249 | 1.076 | 0.004456544 | **0.06016334** |
|  | 17 | MIP-1a | 1.72 | 0.563 | 2.877 | 0.007099557 | **0.06389601** |
|  | 17 | IL-8 | 0.356 | 0.015 | 0.698 | 0.04195742 | 0.28036479 |
|  | 17 | IL-12p70 | 0.686 | -0.007 | 1.378 | 0.051919405 | 0.28036479 |
|  | 16 | MIP-1b | 0.02 | -0.002 | 0.041 | 0.067121871 | 0.28540946 |
| Grip Strength BL-6M | 22 | MIP-1b | 0.133 | 0.052 | 0.213 | 0.003022443 | **0.08160597** |
|  | 22 | MIP-1a | 6.234 | 2.031 | 10.437 | 0.006270464 | **0.08465126** |
|  | 22 | IL-12p70 | 2.613 | 0.68 | 4.547 | 0.01122991 | 0.10106919 |
|  | 22 | PDGF-BB | 0.012 | 0.002 | 0.021 | 0.017076762 | 0.11526814 |
|  | 22 | IL-1RA | 0.038 | 0.003 | 0.073 | 0.03392505 | 0.12365234 |
|  | 22 | IL-2 | 1.468 | 0.126 | 2.81 | 0.033928835 | 0.12365234 |
| Grip Strength BL-1Y | 17 | IL-2 | 2.81 | 0.353 | 5.267 | 0.02836027 | 0.35408544 |
|  | 17 | bFGF | 0.325 | 0.017 | 0.632 | 0.040117651 | 0.35408544 |
|  | 17 | MIP-1a | 6.905 | -0.015 | 13.826 | 0.05043385 | 0.35408544 |
|  | 17 | IL-12p70 | 3.53 | -0.044 | 7.104 | 0.052457102 | 0.35408544 |
|  | 17 | IL-17A | 0.67 | -0.192 | 1.531 | 0.116251688 | 0.62775912 |
|  | 16 | VEGF | -0.014 | -0.035 | 0.008 | 0.183769403 | 0.64942924 |
