# Supplementary material for: Investigating Molecular Signatures Underlying Trapeziometacarpal Osteoarthritis Through the Evaluation of Systemic Cytokine Expression
Source: Front Immunol. 2022 Jan 20;12:794792. doi: 10.3389/fimmu.2021.794792 (PMC8814933; doi:10.3389/fimmu.2021.794792)
Supplement: Supplementary Table 5 — Roles and OA context of cytokines which differentiate patient subgroups. The traditional role of each cytokine or chemokine, as well as well as its associations within OA literature are described. [file Table_5.docx]

**Supplementary Table 5**

| Cytokine | Role | OA Context |
| --- | --- | --- |
| G-CSF, Granulocyte Colony Stimulating Factor | Neutrophil regulation, initiation and maintenance of inflammation[1]. | Higher in plasma of early knee OA patients compared to non-OA controls, unchanged between early and late OA[2]. |
| Il-17a, Interleukin 17 alpha | Acts on a variety of cell-types including synoviocytes, chondrocytes, and immune cells to promote inflammation[3]. | Higher in serum of knee OA patients compared to controls, and positively correlated with radiographic severity[4].  Serum levels are positively and significantly associated with knee cartilage defects, and femoral bone marrow lesions[5]. |
| Il-6, Interleukin 6 | Produced in response to infection or injury, and can act on immune cells as well as synovial fibroblasts; in chronic disease can be over-synthesized and has a pathological inflammatory role[6], can also stimulate release of Il-10, or Il-1Ra to act in negative feedback loop of inflammatory response. | OA serum Il-6 is associated with VAS pain/ depressive state independent of knee OA severity[7].  Women with knee OA experience greater IL-6 reactivity after laboratory evoked pain[8].  Increased knee synovial fluid levels are associated with greater radiographic severity and pain, and knee cartilage loss in older adults[8, 9]. |
| Il-10, Interleukin 10 | Anti-inflammatory, secreted by cells of myeloid and lymphoid lineages and acts to inhibit cytokine production by Th1 cells[10]. | High ratios of Il-6:Il-10 in post-menopausal women have high risk for symptomatic lumbar OA[11].  Patients with knee OA had significantly lower levels of Il-10 in synovial fluid than those with acute meniscal injury[12].  Higher levels in sera of patients with painful knee OA than healthy controls[10]. |
| Il-1b, Interleukin 1 beta | Produced by chondrocytes, mononuclear cells, osteoblasts and synovial tissue and induces production of catabolic and inflammatory factors[13]. | Il-1b in SF of TMJ OA is indicative of increased pain and hyperalgesia[14].  Greater in SF of severe knee OA patients compared to mild/ moderate OA. Greater in serum of healthy controls vs mild/moderate or severe OA patients[15]. |
| Il-12p70, Interleukin 12 phosphorylated subunit 70 | A pro-inflammatory cytokine produced by monocytes, macrophages and dendritic cells, is composed of the combined p35 and p40 subunits and required for the induction of interferon-gamma production which is critical for inducing Th1 cells[16]. | Expressed in synovial lining cells and macrophages in OA and RA patients[17]. |
| Il-2, Interleukin 2 | Required for T-cell differentiation, its activation of T-cells augments production and secretion of other cytokines. Promotes proliferation of B-cells and NK cells and increases cytokine production and cytolytic activity of NK cells[18]. | Concentration of plasma Il-2 in knee OA patients (advanced and early) are higher than healthy controls.  Il-2 is detectable in the synovial membrane of early and late stage knee OA patients, but only in SF within a subset of late stage patients[19]. |
| Il-13, Interleukin 13 | Anti-inflammatory, has been described has chondroprotective in the context of OA[20].  Produced by multiple immune cell types and acts on both immune and non-immune cells to promote fibrosis, and inhibit Th1 driven inflammation as well as Th17 cells[21]. | Similar levels in primary knee OA SF as post-traumatic wrist OA SF[22].  Serum concentration of Il-13 is significantly decreased in advanced knee OA vs early OA[23]. |
| MCP-1, Monocyte chemoattractant protein 1 (also known as CCL2) | Pro-inflammatory, is a key chemokine that regulates the infiltration and migration of monocytes/macrophages, NK and memory T-cells[24]. | Higher in SF than serum of OA patients[25].  Levels of serum MCP-1 (CCL2) were associated with presence and progression of radiographic knee OA and medial joint space narrowing[26]. |
| bFGF, Basic Fibroblast Growth Factor (FGF2) | Binds heparin and heparan sulfate and stimulates processes such as angiogenesis, wound healing, and tissue development[27]. | Plasma and SF bFGF levels were significantly higher in knee OA patients than controls, and correlate with radiographic disease severity[28, 29]. |
| TNFa, Tumor Necrosis Factor Alpha | Pro-inflammatory cytokine which is produced by cells from monocyte/ macrophage lineage and involved in the propagation of the inflammatory response[30, 31]. | Blocking TNFa using adalimumab stopped progression of joint damage in erosive hand OA[32].  TNFa is associated with knee cartilage loss in older adults[8]. |
| IFNy, Interferon Gamma | Primarily secreted by activated T cells and NK cells and can regulate a number of different immune responses including macrophage activation, innate immune system activation, cellular proliferation and apoptosis[33]. | Increased levels of IFNy in synovial fluid of radiocarpal OA patients compared to pre-osteoarthritic and healthy controls.  IFNy mRNA levels in cells derived from temporomandibular joint synovial fluid are higher in OA patients than controls[34]. |
| IP-10, Interferon Gamma Induced Protein 10 (CXCL10) | A chemokine with pro-inflammatory and anti-angiogenic properties which is induced by IFNy, TNFa, NFkb and regulates immune responses through the activation and recruitment of leukocytes[35, 36]. | Plasma and synovial fluid IP-10 levels are inversely correlated with radiographic severity of knee OA[37]. |

**References**

1. Panopoulos AD, Watowich SS: **Granulocyte colony-stimulating factor: molecular mechanisms of action during steady state and 'emergency' hematopoiesis**. *Cytokine* 2008, **42**(3):277-288.

2. Mabey T, Honsawek S, Saetan N, Poovorawan Y, Tanavalee A, Yuktanandana P: **Angiogenic cytokine expression profiles in plasma and synovial fluid of primary knee osteoarthritis**. *Int Orthop* 2014, **38**(9):1885-1892.

3. Onishi RM, Gaffen SL: **Interleukin-17 and its target genes: mechanisms of interleukin-17 function in disease**. *Immunology* 2010, **129**(3):311-321.

4. Chen B, Deng Y, Tan Y, Qin J, Chen LB: **Association between severity of knee osteoarthritis and serum and synovial fluid interleukin 17 concentrations**. *J Int Med Res* 2014, **42**(1):138-144.

5. Wang K, Xu J, Cai J, Zheng S, Yang X, Ding C: **Serum levels of resistin and interleukin-17 are associated with increased cartilage defects and bone marrow lesions in patients with knee osteoarthritis**. *Mod Rheumatol* 2017, **27**(2):339-344.

6. Tanaka T, Narazaki M, Kishimoto T: **IL-6 in inflammation, immunity, and disease**. *Cold Spring Harb Perspect Biol* 2014, **6**(10):a016295.

7. Shimura Y, Kurosawa H, Tsuchiya M, Sawa M, Kaneko H, Liu L, Makino Y, Nojiri H, Iwase Y, Kaneko K *et al*: **Serum interleukin 6 levels are associated with depressive state of the patients with knee osteoarthritis irrespective of disease severity**. *Clin Rheumatol* 2017, **36**(12):2781-2787.

8. Stannus O, Jones G, Cicuttini F, Parameswaran V, Quinn S, Burgess J, Ding C: **Circulating levels of IL-6 and TNF-alpha are associated with knee radiographic osteoarthritis and knee cartilage loss in older adults**. *Osteoarthritis Cartilage* 2010, **18**(11):1441-1447.

9. Nees TA, Rosshirt N, Zhang JA, Reiner T, Sorbi R, Tripel E, Walker T, Schiltenwolf M, Hagmann S, Moradi B: **Synovial Cytokines Significantly Correlate with Osteoarthritis-Related Knee Pain and Disability: Inflammatory Mediators of Potential Clinical Relevance**. *J Clin Med* 2019, **8**(9).

10. Imamura M, Ezquerro F, Marcon Alfieri F, Vilas Boas L, Tozetto-Mendoza TR, Chen J, Ozcakar L, Arendt-Nielsen L, Rizzo Battistella L: **Serum levels of proinflammatory cytokines in painful knee osteoarthritis and sensitization**. *Int J Inflam* 2015, **2015**:329792.

11. Suyasa IK, Kawiyana IK, Bakta IM, Widiana IG: **Interleukin-6 and ratio of plasma interleukin-6/interleukin-10 as risk factors of symptomatic lumbar osteoarthritis**. *World J Orthop* 2017, **8**(2):149-155.

12. Ding J, Niu X, Su Y, Li X: **Expression of synovial fluid biomarkers in patients with knee osteoarthritis and meniscus injury**. *Exp Ther Med* 2017, **14**(2):1609-1613.

13. Kapoor M, Martel-Pelletier J, Lajeunesse D, Pelletier JP, Fahmi H: **Role of proinflammatory cytokines in the pathophysiology of osteoarthritis**. *Nat Rev Rheumatol* 2011, **7**(1):33-42.

14. Alstergren P, Ernberg M, Kvarnstrom M, Kopp S: **Interleukin-1beta in synovial fluid from the arthritic temporomandibular joint and its relation to pain, mobility, and anterior open bite**. *J Oral Maxillofac Surg* 1998, **56**(9):1059-1065; discussion 1066.

15. Heard BJ, Fritzler MJ, Wiley JP, McAllister J, Martin L, El-Gabalawy H, Hart DA, Frank CB, Krawetz R: **Intraarticular and systemic inflammatory profiles may identify patients with osteoarthritis**. *J Rheumatol* 2013, **40**(8):1379-1387.

16. Gee K, Guzzo C, Che Mat NF, Ma W, Kumar A: **The IL-12 family of cytokines in infection, inflammation and autoimmune disorders**. *Inflamm Allergy Drug Targets* 2009, **8**(1):40-52.

17. Sakkas LI, Johanson NA, Scanzello CR, Platsoucas CD: **Interleukin-12 is expressed by infiltrating macrophages and synovial lining cells in rheumatoid arthritis and osteoarthritis**. *Cell Immunol* 1998, **188**(2):105-110.

18. Gaffen SL, Liu KD: **Overview of interleukin-2 function, production and clinical applications**. *Cytokine* 2004, **28**(3):109-123.

19. Scanzello CR, Umoh E, Pessler F, Diaz-Torne C, Miles T, Dicarlo E, Potter HG, Mandl L, Marx R, Rodeo S *et al*: **Local cytokine profiles in knee osteoarthritis: elevated synovial fluid interleukin-15 differentiates early from end-stage disease**. *Osteoarthritis Cartilage* 2009, **17**(8):1040-1048.

20. Wojdasiewicz P, Poniatowski LA, Szukiewicz D: **The role of inflammatory and anti-inflammatory cytokines in the pathogenesis of osteoarthritis**. *Mediators Inflamm* 2014, **2014**:561459.

21. Mao YM, Zhao CN, Leng J, Leng RX, Ye DQ, Zheng SG, Pan HF: **Interleukin-13: A promising therapeutic target for autoimmune disease**. *Cytokine Growth Factor Rev* 2019, **45**:9-23.

22. Teunis T, Beekhuizen M, Van Osch GV, Schuurman AH, Creemers LB, van Minnen LP: **Soluble mediators in posttraumatic wrist and primary knee osteoarthritis**. *Arch Bone Jt Surg* 2014, **2**(3):146-150.

23. Barker T, Rogers VE, Henriksen VT, Aguirre D, Trawick RH, Rasmussen GL, Momberger NG: **Serum cytokines are increased and circulating micronutrients are not altered in subjects with early compared to advanced knee osteoarthritis**. *Cytokine* 2014, **68**(2):133-136.

24. Deshmane SL, Kremlev S, Amini S, Sawaya BE: **Monocyte chemoattractant protein-1 (MCP-1): an overview**. *J Interferon Cytokine Res* 2009, **29**(6):313-326.

25. Stankovic A, Slavic V, Stamenkovic B, Kamenov B, Bojanovic M, Mitrovic DR: **Serum and synovial fluid concentrations of CCL2 (MCP-1) chemokine in patients suffering rheumatoid arthritis and osteoarthritis reflect disease activity**. *Bratisl Lek Listy* 2009, **110**(10):641-646.

26. Longobardi L, Jordan JM, Shi XA, Renner JB, Schwartz TA, Nelson AE, Barrow DA, Kraus VB, Spagnoli A: **Associations between the chemokine biomarker CCL2 and knee osteoarthritis outcomes: the Johnston County Osteoarthritis Project**. *Osteoarthritis Cartilage* 2018, **26**(9):1257-1261.

27. Nugent MA, Iozzo RV: **Fibroblast growth factor-2**. *Int J Biochem Cell Biol* 2000, **32**(2):115-120.

28. Honsawek S, Yuktanandana P, Tanavalee A, Saetan N, Anomasiri W, Parkpian V: **Correlation between plasma and synovial fluid basic fibroblast growth factor with radiographic severity in primary knee osteoarthritis**. *Int Orthop* 2012, **36**(5):981-985.

29. El-Fetiany AE, Kassem EM, El-Barbary AM, Gaber RA, Zyton HA: **Evaluation of plasma basic fibroblast growth factor (bFGF) in primary knee osteoarthritis patients**. *The Egyptian Rheumatologist* 2017, **39**(1):33-37.

30. Feldmann M, Maini RN: **Anti-TNF alpha therapy of rheumatoid arthritis: what have we learned?** *Annu Rev Immunol* 2001, **19**:163-196.

31. Strieter RM, Kunkel SL, Bone RC: **Role of tumor necrosis factor-alpha in disease states and inflammation**. *Crit Care Med* 1993, **21**(10 Suppl):S447-463.

32. Verbruggen G, Wittoek R, Vander Cruyssen B, Elewaut D: **Tumour necrosis factor blockade for the treatment of erosive osteoarthritis of the interphalangeal finger joints: a double blind, randomised trial on structure modification**. *Ann Rheum Dis* 2012, **71**(6):891-898.

33. Tau G, Rothman P: **Biologic functions of the IFN-gamma receptors**. *Allergy* 1999, **54**(12):1233-1251.

34. Vernal R, Velasquez E, Gamonal J, Garcia-Sanz JA, Silva A, Sanz M: **Expression of proinflammatory cytokines in osteoarthritis of the temporomandibular joint**. *Arch Oral Biol* 2008, **53**(10):910-915.

35. Gotsch F, Romero R, Friel L, Kusanovic JP, Espinoza J, Erez O, Than NG, Mittal P, Edwin S, Yoon BH *et al*: **CXCL10/IP-10: a missing link between inflammation and anti-angiogenesis in preeclampsia?** *J Matern Fetal Neonatal Med* 2007, **20**(11):777-792.

36. Lee EY, Lee ZH, Song YW: **CXCL10 and autoimmune diseases**. *Autoimmun Rev* 2009, **8**(5):379-383.

37. Saetan N, Honsawek S, Tanavalee A, Tantavisut S, Yuktanandana P, Parkpian V: **Association of plasma and synovial fluid interferon-gamma inducible protein-10 with radiographic severity in knee osteoarthritis**. *Clin Biochem* 2011, **44**(14-15):1218-1222.
